# Supplementary material for: Population Genetics of California Gray Foxes Clarify Origins of the Island Fox
Source: Genes (Basel). 2022 Oct 14;13(10):1859. doi: 10.3390/genes13101859 (PMC9602142; doi:10.3390/genes13101859)
Supplement: Supplementary file 1 [file genes-13-01859-s001.zip › SupplementaryFigures-rev.pdf]

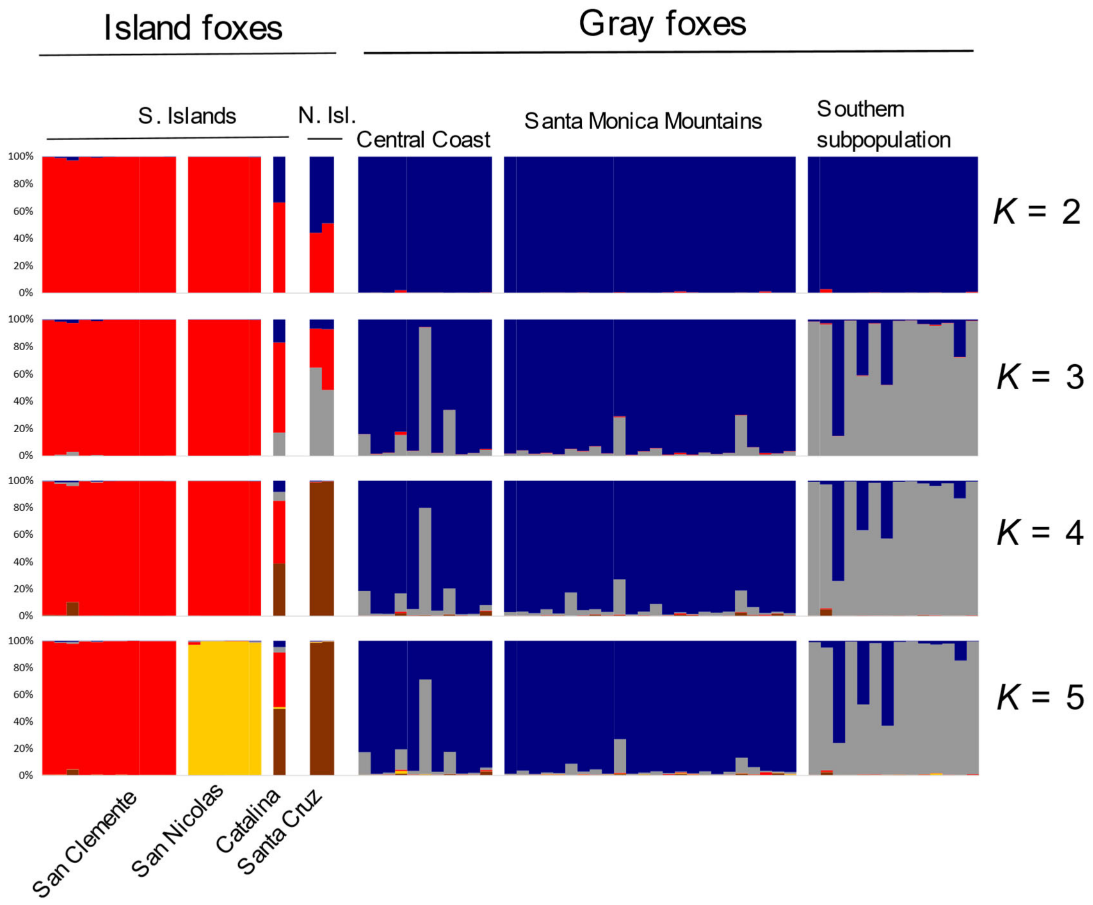

Supplementary Figure S1. Admixture analysis of 69 western gray and island foxes from the Central Coast, Santa Monica Mountains, Southern subpopulation, and three Channel Islands with 20 microsatellite loci conducted in program Structure, assuming K = 2, 3, 4, and 5 clusters. At K = 2, results are the same as in the full analysis (see main text, Figure 2). At K = 3, the southern subpopulation splits from the Santa Monica Mountain and Central Coast populations. At K = 4, all island foxes are distinct from gray foxes and northern and southern islands (except for Catalina) become distinct. At the highest level of resolution (K = 5), all islands cluster as distinct except Santa Catalina, possibly because only one sample was represented and possibly due to recent translocations from the northern islands. Contrary to the corresponding analysis using all populations, the island foxes from the northern island (Santa Cruz) and Santa Catalina Island, cluster more closely with the southern subpopulation than with the Santa Monica Mountain population. Log probability of the data (SD) for *K* = 1–10 were as follows: -4185 (1.06), -3534 (4.96), -3489 (9.45), -3539 (220.3), -3338 (117.76), -3516 (24.11), -3774 (307.91), -3522 (20.38), -3614 (111.9), and -3496 (221.61),.

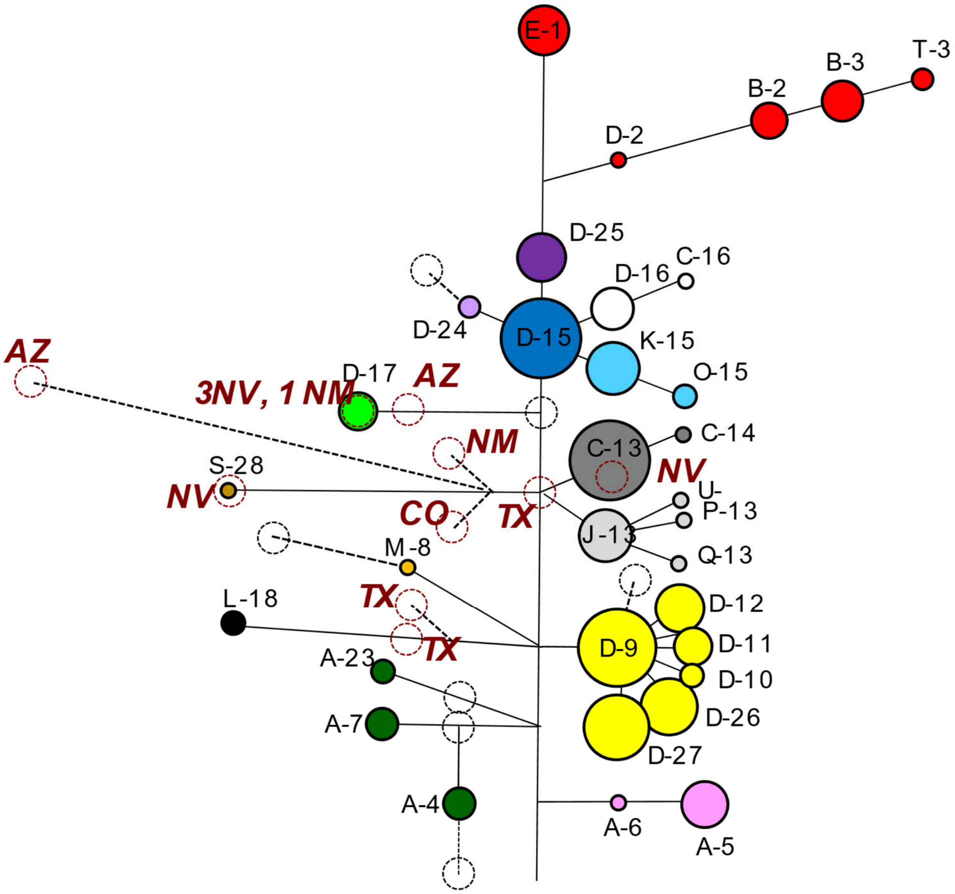

Supplementary Figure S2. Median-joining network composed of 785 bp of cytochrome b and D loop sequence, including 345 foxes from the current study along with all haplotypes observed in two other studies. Branch lengths are proportional to the number of substitutions and node sizes are proportional to the number of samples in the present study. Dashed branches and circles represent connections to haplotypes (nodes), respectively, presented in the data first presented by Hofman et al. [8] (black, n = 186) and Reding et al. [7] (maroon, n = 16). Orthologous haplotypes from Hofman et al. [8] and Reding et al. [7] were excised from complete mitogenomes. All haplotypes from Hofman et al. were collected in California, whereas those from Reding et al. are annotated in maroon text as follows: Arizona (AZ), Colorado (CO), Nevada (NV), New Mexico (NM), and Texas (TX).
