# Supplementary material for: Population Genetics of California Gray Foxes Clarify Origins of the Island Fox
Source: Genes (Basel). 2022 Oct 14;13(10):1859. doi: 10.3390/genes13101859 (PMC9602142; doi:10.3390/genes13101859)
Supplement: Supplementary file 1 [file genes-13-01859-s001.zip › TableS2-primers-rev.pdf]

Supplementary Table S2. Primer sequences for 20 loci used for gray foxes (*Urocyon cinereoargenteus*) and island foxes (*U. littoralis*), along with reaction concentrations (same for forward, F, and reverse, R, primers), fluorescent dyes, multiplex sets, species for which primers were originally designed, and references.

Set	Locus	Conc. (µM)	Dir.	Dye	Primer sequence	Species	Ref.
1	CXX-402	0.42	F	PET	GGGTAATTCATCCAGTGCCTT	*V. vulpes*	a
			R		TATGCAAACATGCAAACATGC	*V. vulpes*	a
1	AHT-142	0.50	F	PET	AAGCAGATCCTAGAGCAGCA	*V. vulpes*	a
			R		CCCCACAGTTTAGAAATATCTGC	*V. vulpes*	a
1	REN105L03	0.42	F	VIC	GGTGCCTGACAAGATGGAAT	*V. vulpes*	b
			R		GAGATTGCTGCCCTTTTTACT	*V. vulpes*	b
1	CPH18	0.42	F	6FAM	CAGAGATACGTCTTGACACTAGCAGA	Dog	c
			R		GGCCATTCCTGAAACACTTG	*V. vulpes*	b
1	CPH8	0.84	F	NED	TGCTCAATTGATTAGTCCGTCT	*V. vulpes*	b
			R		TAGATTTGATACCTCCCTGAGTCC	Dog	c
1	REN54P11	0.42	F	PET	TCCCTCTGGCTCAGTTAGAA	*V. vulpes*	b
			R		ATCAGCATCACCTGGGAACA	*V. vulpes*	b
1	FH2001	1.00	F	6FAM	TCCTCCTCTTCTTTCCATTGG	Dog	d
			R		TGCATTCTTCTTGCATGCTT	*V. vulpes*	b
2	CPH2	0.67	F	NED	TTCTGTTGTTATTGGCACCA	*V. vulpes*	b
			R		TTCTTGAGAACAGTGTCCTTCG	Dog	c
2	FH2088	0.17	F	VIC	CCCTCTGCCTACATCTCTGC	Dog	d
			R		TAGGGCATGCATATAACCAGC	Dog	d
2	REN162C04	0.50	F	VIC	AAAATCAGCATTATTCTTTCCACA	*V. vulpes*	b
			R		TGGCTGTATTCTTTGGCACA	Dog	e
2	AHTh-171	0.59	F	PET	AGGTGCAGAGCACTCACTCA	Dog	f
			R		CCTCAAACCCAGGTGAAGC	*V. vulpes*	b
2	INU055	0.50	F	NED	CCAGGTGTCCCTATCCATCT	*V. vulpes*	b
			R		GCACCACTTTGGGCTCCTTC	Dog	g
2	FH2010	0.25	F	6FAM	AAATGGAACAGTTGAGCATGC	Dog	d
			R		CCCCTTACAGCTTCATTTTCC	Dog	d
2	FH2004	0.17	F	VIC	CTAAGTGGGGAGCCTCCTCT	Dog	d
			R		ACTGTGACCTACTGAGGTTGCA	Dog	d
3	CXX0279	0.50	F	PET	TGCTCAATGAAATAAGCCAGA	*V. vulpes*	b
			R		GAATTTGATTACTCTGGAAAACTCC	*V. vulpes*	b
3	INU030	0.42	F	6FAM	GGCTCTGTGCTCAAGTCTGT	*V. vulpes*	b
			R		CATTGAAAGGGAATGCTGGT	Dog	g
3	AHT-137	0.50	F	VIC	TACAGAGCTCTTAACTGGGTCC	Dog	h
			R		CCTTGCAAAGTGTCATTGCT	Dog	h
3	FH2054	0.25	F	NED	GCCTTACTCATTGCAGTTAGGG	*V. vulpes*	b
			R		ATGCTGAGTTTTGAACTTTCCC	Dog	d

3	C01.424	0.25	F	PET	AGCTTAGCTTACTGCCCTGG	Dog	i
			R		TCCTTTGGTTTTTAGCAGGG	Dog	i
3	C08.618	0.42	F	6FAM	CAACCCAGGGTGGAAGC	Dog	i
			R		GCCCAGAATCCATTGAGAAA	*V. vulpes*	b

References

a - Wandeler P, Funk SM (2005) Short microsatellite DNA markers for the red fox (Vulpes vulpes). Molecular Ecology, 6, 98-100.

b - Moore M., S. K. Brown, B. N. Sacks. 2010. Thirty-one short red fox (Vulpes vulpes) microsatellite markers. Molecular Ecology Resources 10:404-408.

c - Fredholm M, Winterø AK (1995) Variation of short tandem repeats within and between species belonging to the Canidae family. Mammalian Genome, 6, 11-18.

d - Francisco L.V., Langston A.A., Mellersh C.S., Neal C.L. & Ostrander E.A. (1996) A class of highly polymorphic tetranucleotide repeats for canine genetic mapping. Mammalian Genome 7, 359–62.

e - Breen M, Jouquand S, Renier C, et al (2001). Chromosomespecific single-locus FISH probes allow anchorage of an 1800-marker integrated radiation-hybrid/linkage map of the domestic dog genome to all chromosomes. Genome Research, 11,1784-1795.

f - Ostrander EA, Sprague GF, Rine J (1993) Identification and Characterization of dinucleotide repeat (CA)n markers for genetic mapping in dog. Genomics, 16, 207-213.

g - Ichikawa Y, Takahashi S, Tsumagari M, et al (2002) Identification a 112 nd characterization of 40 dinucleotide microsatellites in the dog genome. Animal Genetics, 3, 400-402.

h - Holmes NG, Dickens HF, Parker HL Binns MM, Mellersh CS, Sampson J (1995) Eighteen canine microsatellites. Animal Genetics, 26, 132-133.

i - Ostrander, E.A., Mapa, F., Yee, M., Rine, J. (1995). One hundred new simple sequence repeat-based markers for the canine genome. Mamm. Genome 6, 192-195.
